# Comparison between rigid and soft poly-articulated prosthetic hands in non-expert myo-electric users shows advantages of soft robotics

**DOI:** 10.1038/s41598-021-02562-y

**Published:** 2021-12-14

**Authors:** Patricia Capsi-Morales, Cristina Piazza, Manuel G. Catalano, Giorgio Grioli, Lisa Schiavon, Elena Fiaschi, Antonio Bicchi

**Affiliations:** 1 Istituto Italiano di Tecnologia, Via Morego 30, 16163 Genoa, Italy; 2 Centro “E. Piaggio” and Dipartimento di Ingegneria dell’Informazione, University of Pisa, Largo Lucio Lazzarino 1, 56127 Pisa, Italy; 3 Technical University of Munich, Boltzmannstr. 3, 85748 Garching, Germany; 4 Department of Translational Research and New Technologies in Medicine and Surgery, University of Pisa, Pisa, Italy; 5 Operational Unit of Recovery and Functional Rehabilitation, Usl Toscana NordOvest, Via Firenze 48, 54033 Marina di Carrara, Italy

**Keywords:** Rehabilitation, Biomedical engineering, Mechanical engineering

## Abstract

Notwithstanding the advancement of modern bionic hands and the large variety of prosthetic hands in the market, commercial devices still present limited acceptance and percentage of daily use. While commercial prostheses present rigid mechanical structures, emerging trends in the design of robotic hands are moving towards soft technologies. Although this approach is inspired by nature and could be promising for prosthetic applications, there is scant literature concerning its benefits for end-users and in real-life scenarios. In this work, we evaluate and assess the role and the benefits of soft robotic technologies in the field of prosthetics. We propose a thorough comparison between rigid and soft characteristics of two poly-articulated hands in 5 non-expert myo-electric prosthesis users in pre- and post-therapeutic training conditions. The protocol includes two standard functional assessments, three surveys for user-perception, and three customized tests to evaluate the sense of embodiment. Results highlight that rigid hands provide a more precise grasp, while soft properties show higher functionalities thanks to their adaptability to different requirements, intuitive use and more natural execution of activities of daily living. This comprehensive evaluation suggests that softness could also promote a quick integration of the system in non-expert users.

## Introduction

Hands play an important role in human life for prehensile, proprioceptive, and communication purposes. Compensating for losing fine and coordinated function of upper extremities with prostheses is a medical, technological, psychological, and social challenge^1^. Even though artificial limbs open up the prospect of restoring some missing capabilities, there is still a wide gap between available commercial devices and the perceived demands of prosthesis users^2^.

Current commercial solutions go from simple body-powered systems to more advanced self-powered rigid hands, commonly anthropomorphic in appearance. The former are most typically hook-like grippers, controlled using a shoulder harness. The latter group, also referred to as *poly-articulated*, *multi-fingered*, or *bionic hands*, presents a number of links and joints similar to the human hand, although not all of them may be independently driven by one or more motors. Sometimes they include elastic elements, such as springs, mainly used to reopen the hand. Some examples are the Michelangelo hand^3^, Bebionic hand^4^, i-Limb hand^5^, Taska hand^6^, Mia hand^7^ and Vincent hand^8^. The state-of-the-art hands are “rigid”, meaning that, once the position of motor(s) is fixed, the position of all links and joints is fixed as well. Self-powered prostheses can be commanded in several ways, ranging from simple switches to more advanced techniques that use surface or implantable electromyographic sensors (EMG, IMES) and/or inertial measurement units (IMU). Most myo-electric commercial hands associate the prosthesis movement to the user’s muscles activation using only 2 sEMG sensors, and allow the selection of the grip pattern through switching techniques.

Just like humans modify their hands to match different task requirements (e.g. wearing gloves to increase hand traction, or using specialized tools), each prosthetic technology could favour the execution of specific activities and may be preferred depending on the context and situations. Thanks to their simplicity and ruggedness, hooks are well suited for precision grips^9^ and for coarse environments^10^. Body-powered systems also provide valuable sensory feedback, which makes them still effective despite their simplicity. This was confirmed in the Cybathlon competitions of 2016 and 2020, where both Powered Arm Prosthesis Races were won by a body-powered device (a hook^9^ and a hand^11^, respectively). By contrast, myo-electric prostheses are less cumbersome and offer more dexterous capabilities, so that they are preferred for light duties, office work, and social outing^12^.

Unfortunately, the rejection rate of upper limb prostheses is high among unilateral amputee subjects^13^, and a large part of the users rely mostly on the contralateral limb. While several studies use the length of daily use as an indicator of the success^14,15^, others focus on additional factors such as functionalities and patient satisfaction^12^. The initial use of artificial limbs is not intuitive^16^ and an adequate training program conducted by specialized clinicians is extremely valuable for acceptance^17–19^. A study^20^ proved that sufficient therapeutic training leads to significant improvement in efficiency and skill, while it had a smooth effect in spontaneity. Spontaneity (i.e. use of the prosthesis without conscious effort^21^) seems to be related with the sense of embodiment, and may be influenced by the mechanical properties of the system and its perceived function.

The acceptance of an artificial hand is influenced by many aspects, but important reasons for rejection are poor functionality and intuitiveness^22^. In the last decade, soft robotics technologies became an alternative approach in the design of robotic hands^23^ that favours adaptivity, robustness and pleasant interaction with people. Composed of soft parts and flexible joints, these hands take inspiration from biology and promote anthropomorphic characteristics beyond mere aesthetics. The growing interest in these technologies is evident in the field of human–robot interaction, but it is also emerging in prosthetics^24–27^. The grasp patterns in soft hands are defined in response to the contact with objects and the surrounding environment. This characteristic simplifies system control while keeping an advanced hand functionality. Although the force output of state-of-the-art “soft hands” is improving, they are usually behind rigid hands, especially for continuum soft robotic designs. However, in most of the common activities of daily living, objects weight around 1-2 kg, which belong or are close to the range of forces that can be applied with available advanced soft hands. In our protocol, we included objects with different weights. Even though the application of soft-robotics technologies to prosthetics may be beneficial^28^, only few studies in literature validate soft-robotics with real prosthesis users (e.g.^29,30^ for continuum soft prostheses and^31–33^ for articulated soft prostheses).

In this article, we explore the role that the use of soft-robotics technology in the design of bionic limbs plays toward functionality and usability. We hypothesize that (a) rigid fingers can be more suitable for precise grasping while soft hands may favour adaptability and a more natural use in advanced tasks, while (b) soft properties may result more intuitive and promote substantial learning in limited time and (c) enhance the sense of embodiment in users.

**Figure 1:**
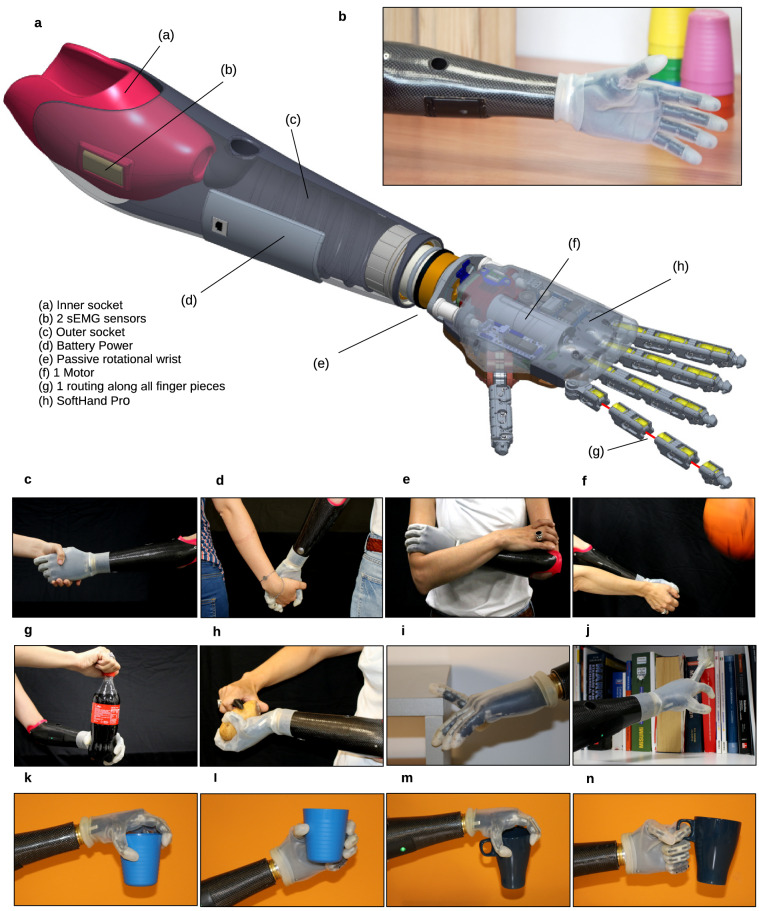
SoftHand Pro system overview. The hand is composed of three main components: a myoelectric poly-articulated prosthetic hand with soft properties, a passive rotational wrist module, and an outer socket embedding the inner socket for the stump of each user, containing a myoelectric interface/controller. (**a**) Systems architecture: The motor and the motor control boards are embedded (f) within the myoelectric hand (h), whereas the sEMG-based (b) myoelectric interface/controller (d) is housed within the socket (a, c). A unique tendon (g) connects all the pieces of the fingers (19 DoF) and several elastic bands to a motor (1 DoA) (f), creating a soft under-actuated system designed with the concept of synergies. The passive rotational wrist (e) is placed between these two modules. (**b**) View of the real system with its glove to increase the fingers traction when grasping. Examples of the hand characteristics in use: (**c**–**f**) soft interaction with other subjects or the user own body, (**g**–**h**) bimanual activities, (**i**–**j**) safe deformation of the fingers in contact with external forces and (**k**–**n**) the system adaptive capability to grasp the same object with different pattern grasps depending to the grasping approach decided by the user.

By using standard assessment protocols to measure hand functionality and user-perception, we compare the experience of a group of prosthesis users with examples of excellence of rigid and soft poly-articulated hands. For the group of rigid hands, we used two of the most advanced hands, the i-Limb and the Bebionic hands, which are commercially available and widely tested. Because there are no commercially available soft prosthetic hands, as a representative of this group, we adopted an advanced research prototype developed in our laboratory, the SoftHand Pro (SHP) (see Fig. 1 and the “Material and methods” section). The rationale for this choice is the high TRL demonstrated by the SHP, which has been tested with few tens of patients in at least 10 different institutions worldwide (e.g.^31,34,35^).

Considering the non-expert myoelectric users condition of our participants, we investigate the relation between the progress rate in (a) manipulation proficiency and (b) the acceptance of the technology, with the two different types of hands. The authors believe that this study contributes to an understanding of the different effects that specific design principle have on several aspects of prosthesis use and acceptance. In doing so, the paper methodology also illustrates the role that training has in using very different prosthetic technologies.

## Results

### Functional assessment

**Figure 2:**
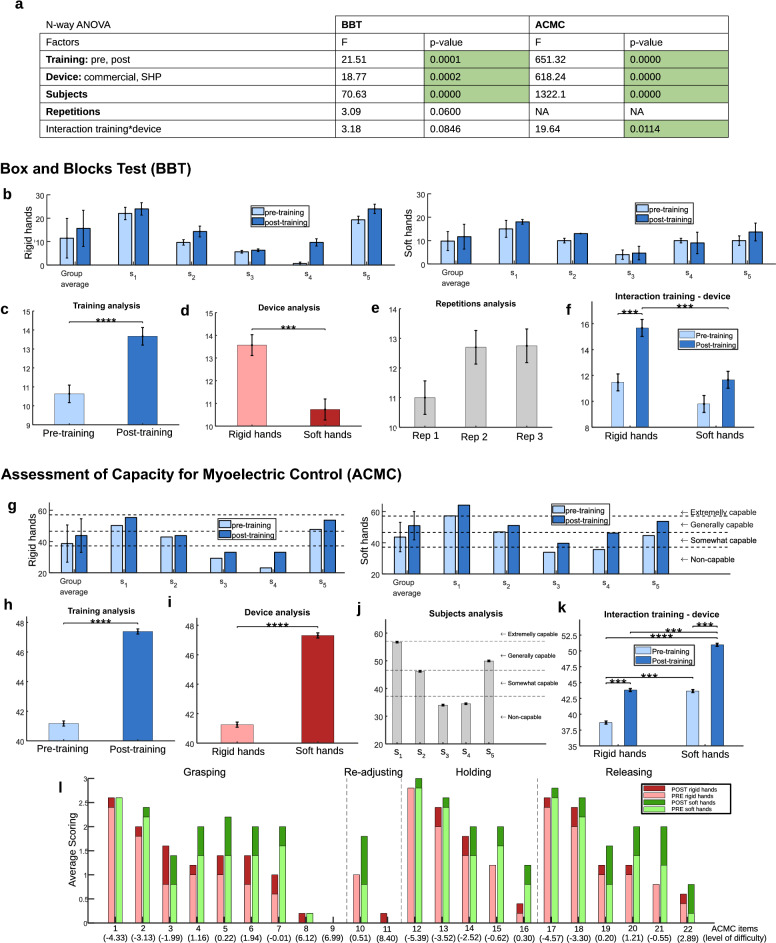
Functional assessment. (**a**) Shows a table with the statistical results for each assessment and factor included. (**b**) BBT score (n = 3 repetitions for each subject s_i_). (**c**–**f**) Present the relation between the factors analysed, including an interaction test between training and the device. (**g**) Shows results of ACMC for each subject and the group average. (**h**–**k**) Present the relation between the factors analysed, including an interaction test between training and the device. After a post-hoc Tukey test, significance is expressed with asterisks in a GraphPad style among bars, when occurs. *P* values < 0.001 are summarized with *** and *p* < 0.0001 is expressed with ****. (**l**) Details the average item score for each hand in pre- and post-training conditions.

To evaluate the performance and functionalities of the two systems we considered 2 standard assessments, the Box and Blocks test (BBT) and the Assessment for Capacity of Myoelectric Control (ACMC). Figure 2 reports the data and the statistical analysis for both assessments. Figure 2a details the statistical results of the BBT from an n-Way ANOVA test with significance in all factors studied, except for repetitions and the interaction test between training and device. The participant improved their performance within the 3 repetitions (+ 15.68% after the first repetition, Fig. 2e)—for this reason, we considered repetition as a factor inside of the n-Way ANOVA test. Figure 2b presents the scores from the BBT, including the average of the 3 repetitions performed by each subject and its standard deviation for pre- and post-training conditions with both rigid and soft systems. The average of the group is reported for each prosthetic hand. The rigid commercial hands obtained a score of 11.47 ± 8.46 and 15.57 ± 7.70, while the soft hand got 9.8 ± 4.04 and 11.67 ± 5.31, pre- and post-training, respectively. Figure 2c–f present the estimated mean and standard error for different aspects of the study: the training effect, device, learning between repetitions and the interaction between training and device used. After applying the post-hoc test (details in “Material and methods” section) to prove differences among aspects inside of a factor, Fig. 2c evidences the benefit of the therapeutic training, which allows the participants to improve their performance by 28.6%. Figure 2d shows better results with the rigid devices for BBT, while the performance with soft hands degrades by 20.93%.

The ACMC includes more functional tasks and different types of grasps for the manipulation of various objects. Data extracted from the ACMC results are presented in Fig. 2g–l. Figure 2g shows a general improvement of the ACMC score after training for all subjects and both devices. The average values for the rigid hands are 38.68 ± 11.90 and 43.82 ± 10.74, pre- and post-training, respectively. Likewise, the averages for the soft hand scores are 43.66 ± 9.43 and 50.96 ± 9.08. Figure 2a reports the statistical results for the ACMC test with significance in training, devices, and subjects. The analysis of factors proves the positive effect of training (Fig. 2h) with a score of 41.17 (pre-training) and 46.98 (post-training). Results in Fig. 2i show better performance for the soft hand, with 41.25 (Rigid) and 46.90 (Soft). Figure 2j proves the different capabilities from the participants, with none being considered *extremely capable* in the clinical interpretation of ACMC, which reinforces their non-expert condition in myo-electric control. Their performance tendency corresponds to the one observed in BBT scores, in Fig. 2b. The interaction test in Fig. 2k proves a similar level of learning for both hands properties (rigid and soft), but a higher starting functionality in soft hands. Note that the score for rigid hands post-training is not significantly different from the score for soft hands pre-training. The analysis of the individual scoring for the 22 items of ACMC allows to extract additional information from this test. Figure 2l reports the average score per item in each experimental condition. It is possible to observe a training effect related to the characteristics of the prostheses depending on the item, even though there is a general consistency in learning (see Fig. 2k). Differences between training conditions per system evidences the items in which has been a larger learning during the training sessions. We observe a larger learning in intermediate difficulty items. Among them, rigid hands favoured (with + 0.5 points) the learning of: 3—*Precision grip, without support* and 6—*Grasping timing*. Note that notable learning occurred when items can be favoured by hand properties and there are opportunities for enhancement w.r.t the starting condition. On the other hand, soft hands favoured (with + 0.5 points) the learning of: 3—*Precision grip, without support*, 4—*Appropriate grip force*, 5—*Grasping in different positions*, 6—*Grasping timing*, 10—*Repetitive grip & release*, 14—*Holding in motion*, 19—*Releasing in different positions*, 20—*Releasing timing*, 21—*Coordinating both hands in release* and 22—*Release without visual feedback*.

### User-perception

**Figure 3:**
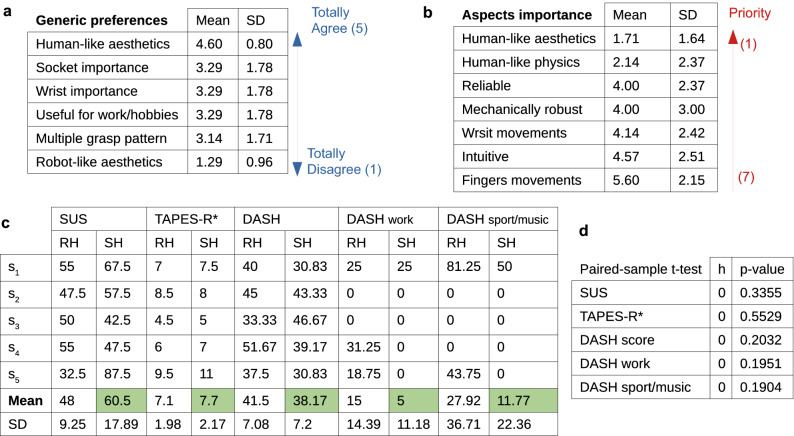
User-perception survey results. (**a**) Shows participants general preferences and (**b**) priorities in prostheses, collected before testing the systems. (**c**) Summarize user-perception scores post-training. RH and SH refer to rigid and soft hands, respectively. In SUS and TAPES-R*, the higher score indicates larger device acceptability and user satisfaction. Contrarily, in the DASH, the lower score indicates less difficulties perceived by the user to perform the included activities (0 represents the minimum score). The higher average positive user-perception is highlighted in green for each survey. (**d**) Presents the statistical analysis for user-perception surveys.

Figure 3a shows the preconception and Fig. 3b the priorities of the participants. Regarding generic preferences, the participants disagree with a *robot-like aesthetics* design, while almost all agree with the importance of *human-like aesthetics*. Similarly, *human-like aesthetics* and *human-like physics* are the aspects with a higher priority for a desirable device. The lowest priority is given to the *independent finger movements*, which could be related to poor expectations because of their non-expert myo-electric users’ condition.

The results of the post-training surveys are presented in Fig. 3c. For both SUS and TAPES-R* tests, a higher score implies better acceptability and satisfaction of the device. The soft hand obtained better performance with an average score of 60.5 ± 17.89 in SUS and 7.7 ± 2.17 in TAPES-R*. The latter is close to the score obtained by rigid hands, equal to 7.1 ± 1.98. In the DASH test, the lower score corresponds to higher perceived functionality, as the scale range from “no difficulty perceived” (minimum) to “unable to perform the task” (maximum). As shown in Fig. 3d, DASH scores suggest a higher perceived functionality for the soft hand (38.17 ± 7.2). This test presents two optional sections that focus on abilities in working conditions and hobbies. There, the soft hand is perceived with a similar or better functionality compared to rigid hands, presenting a score of 5 ± 11.18 and 11.77 ± 22.36 for those subsections, respectively. Although the average values favour soft properties, no statistically significant deviance was found between hands on surveys after a paired-sample t-test (Fig. 3d). A larger sample of participants is required to further explore user-perception preferences because of inter-subject variability.

### Embodiment assessment

**Figure 4:**
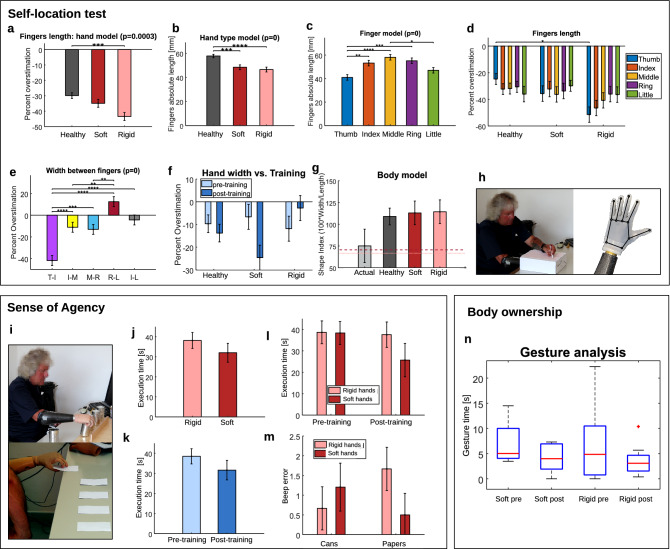
Embodiment assessment. Significant *p* values obtained from n-Way ANOVA test for self-location and sense of agency tests are included in figures title. After a post-hoc Tukey test, significance is expressed with asterisks among bars, when occurs. Significant *p* values < 0.01 are expressed with **, *p* < 0.001 with *** and *p* < 0.0001 with ****. (**a**–**d**) Present the analysis of the fingers length with the corresponding estimated means and standard errors of each group. Data are presented as percent of overestimation and absolute values. (**e**–**f**) Shows the analysis of the width estimation and its relation with training for each system, respectively. (**g**) Presents the distortion on the aspect ratio among hands, where “actual” refers to the anthropomorphic shape index of the subjects. The dashed and dotted lines refer to the real aspect ratio of the soft hand (SHP) and the rigid hand (i-limb), respectively. (**h**) Shows images of the experimental setup and the landmarks asked to mark. (**i**–**m**) Presents the results of the sense of agency test. All cases where the action was successfully performed—moving 5 cans or turning 5 card in less than 1 min were considered in the analysis of execution time. All cases where the subjects gave a valid response about the counted beeps were considered in the analysis of the beeps error. (**n**) Presents the body ownership test for s_5_, with information about the gesture time.

This protocol evaluates the three main components that describe the sense of embodiment^36^: (a) the self-location, (b) the sense of agency and (c) the body ownership. The *self-location test* evaluates the physical perception of each device, applying the method used in^37^. Figure 4 shows the results with amputee subjects, where the healthy hand model obtained (see Fig. 4a) a similar underestimation as in literature [M = − 27.9%]. However, the analysis of both prosthetic devices showed a higher underestimation of the missing hand. The results proved a better reconstruction for the soft hand, with a statistically significant difference only between the healthy and the rigid hand. We also evaluated differences in terms of absolute values of the fingers length, presented in Fig. 4b, where the body model of the healthy hand differs significantly from both prosthetic aids. The authors in^37^ observed 3 clusters in fingers length: the thumb, the index-middle fingers, and the ring-little fingers. As visible in Fig. 4c, the middle finger results significantly different from the thumb and the little finger, while the index and the ring are in an intermediate position, still different from the thumb. The percentage of overestimation for each hand is presented in Fig. 4d (*p* = 0.6429). In agreement with^37^, the fingers length in the healthy hand model is increasingly underestimated from the thumb to the little finger. An opposite situation occurs for the two prosthetic systems, with a larger variation for the rigid hand with respect to healthy hands. To assess the perceived hand width model, the distance between pairs of adjacent knuckles was computed. Results in^37^ showed a strong overestimation [M = 68%] with a moderate overestimation observed for the thumb-index knuckles. Contrarily, our results (see Fig. 4e) showed underestimation for most of the segments, with the largest value in thumb-index knuckles. Similar values were observed for all hands width model in pre-training (Fig. 4f), but results suggest a strong effect after training depending on the prosthesis tested (*p* = 0.0539). To assess the overall hand shape, the Napier’s shape index was adopted^37^, which quantifies its aspect ratio. Results in the literature show a massive overestimation of width relative to the length, where *actual* was around 60 and *healthy* around 150. The results for prosthesis users (Fig. 4g, *p* = 0.3825) showed a larger *actual* index, and a lower perception of the *healthy* hand, the *soft* and the *rigid* hand shape indices (around 115).

The *sense of agency test* includes a motor-cognitive dual-task. Figure 4j–m presents the execution time of the action (motor task) for successful cases and the number of errors while counting beeps (cognitive task). An n-Way ANOVA test was used for different factors (prosthetic systems, pre- and post-training conditions and the motor task executed) but no statistically significant difference was found, probably because of the limited number of participants. Regarding the execution time (*p* = 0.3443), Fig. 4j suggests that the soft hand performed faster than the rigid hand. Figure 4k indicates improvement after training (*p* = 0.2831), and Fig. 4l presents the interaction between systems and training (*p* = 0.3704). Concerning the cognitive task, Fig. 4m shows the error while counting the number of beeps between hands for each manipulation task (*p* = 0.1485). While rigid hands obtained a lower rate of errors in the moving cans task, the soft hand showed higher multi-tasking capability in the turning cards task.

The *body ownership test* consists of observing the use of the prosthesis in spontaneous gestures. Despite 96 verbal descriptions were done by the 3 subjects, only one user presented at least one gesture with the prosthesis for each object. Data of the 32 descriptions for the most dexterous user, with both prosthetic systems in pre- and post-training conditions, are presented in Fig. 4n. Although there is not a clear preference between the two devices, results show lower median values and a smaller variability after therapeutic training. Observations from the other two subjects are included in the “Discussion” section.

## Discussion

Functional assessments prove that, while rigid hands showed better performance in the BBT (Fig. 2d), the soft hand obtained higher scores in the ACMC. In the BBT, the reduction of compensatory movements could promote soft prostheses, but generally, conventional rigid hands allow a faster response for this task. Contrary, in the ACMC, which gives a more extensive evaluation of a hand functionality as it is based on clinical observations during gripping, holding and releasing actions, the soft hand proved to be a more functional system and promising alternative to commercial rigid hands for many reasons related to their adaptability. Further investigations point toward the role of compliant wrists on soft prosthetic arms during the execution of compensatory movements, which was out of the scope of this paper.

**Figure 5:**
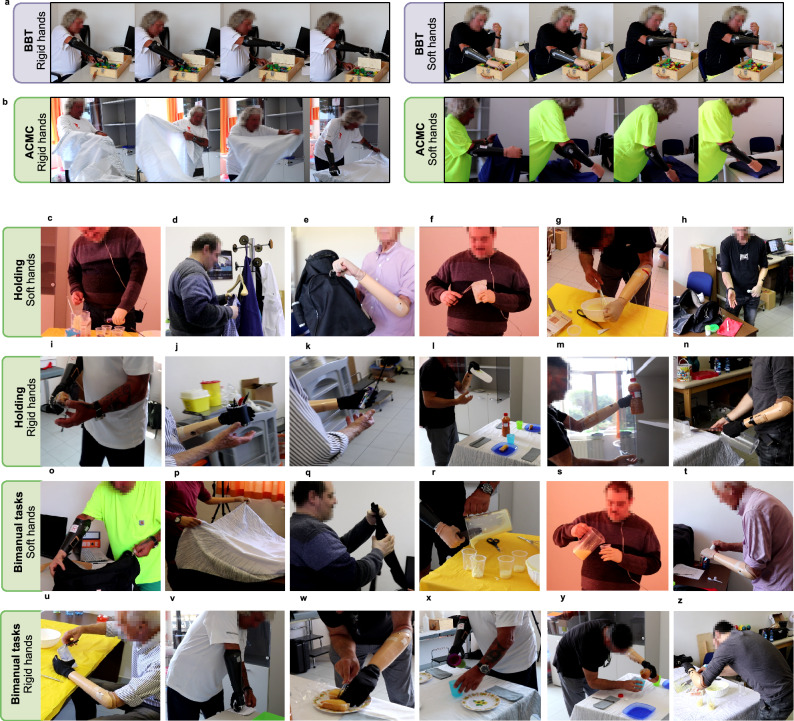
Functional assessment examples. (**a**) Shows a participant executing the BBT with the rigid hand (Bebionic hand) and with the soft hand (SoftHand Pro). (**b**) Presents clothes manipulation during the ACMC test, performed with both rigid and soft hands by the same user. (**c**–**z**) Expose examples of the ACMC test. Frames from all participants performing activities with rigid and soft prostheses.

As shown in Fig. 5a, rigid hands allow participants to achieve more repetitive and precise grasps, presenting only two fingertips in contact with the block. Indeed, even though without significance (Fig. 2a), the interaction test suggested a larger improvement after training with the rigid hands in the BBT (Fig. 2f). Nonetheless, the lack of adaptability leads to larger compensatory movements for rigid hands, as suggested by the shoulder position in the photo-sequence of Fig. 5a. Contrarily, softness permits interaction with multiple contact points when grasping, and enhances a more natural body posture (see Fig. 5a). Despite this, participants relied more on visual feedback when using soft hands, suggesting that the lack of sensory feedback could cause a higher grasp uncertainty.

Figure 2g shows a higher ACMC score on average for the soft hand, probably thanks to its adaptability to different situation requirements and objects. ACMC results suggest a consistent improvement after training (about 7 points) with both rigid and soft systems (Fig. 2k). Figure 5b presents a subject doing clothes manipulation with both soft and rigid prostheses. Even if encouraged by the experimenter to exploit hand functionalities, the subject uses the rigid hand only as an extension of the stump but actively uses the soft hand to complete the task. In addition, despite rigid poly-articulated commercial hands give the possibility to switch among grasping patterns, we did not observe any intentional selection of different hand configurations during the assessment (not even during post-training sessions). If it occurred, it was by error and disrupted the flow of the action. Note that non-expert myoelectric users could experience fatigue in using switching techniques due to difficulties in performing individual muscle contraction. Subjects may require extensive training to incorporate this feature in their ADL.

In the ACMC, the analysis of the scoring per item highlighted larger learning (after only 4 h of training) with the soft hand in many aspects. Among those, items as 4, 10, 14, 20 and 22 (Fig. 2l), which are related to intuitive use and reliability on the device. As shown in Fig. 5c–f, soft technologies promote a more natural posture, that increases control capabilities and grasp reliability during the holding phase (Fig. 5c–e). Some participants even performed a secondary action with the contralateral hand while holding the primary object with the soft hand (Fig. 5f–g) or feel confident in speaking while executing a task (Fig. 5h). Contrarily, Fig. 5i–m show that most of the participants did not feel confident in holding and moving objects with the rigid devices. This issue could be related to grasp reliability or to poor control skills. Nonetheless, users with higher control expertise could still present a reliable holding, as in Fig. 5n. Items 5, 19 and 21 (Fig. 2l), which also present a faster improvement between pre- and post-training conditions for soft properties, are related to more natural coordination with the contralateral arm. We observe more dexterous coordination during bimanual activities with the soft hand. Thanks to the softness and the adaptability of the SHP (Fig. 5o–t), users experience a safer and more intuitive grasp. This is evident when performing advanced activities, such as closing a soft bag (Fig. 5o) or folding clothes (Fig. 5p–q), precise actions (Fig. 5r) or holding fragile objects without support (Fig. 5s–t). In the latter, proper control of the force applied is fundamental for the success of the action. In contrast, most of the participants used the rigid device only as firm support to anchor objects (Fig. 5u–w). The poor adaptability of rigid hands can force the arm configuration into a more uncomfortable body position (Fig. 5x–z).

Giving the small number of participants and the variability among subject preferences, it is not possible to extract statistically significant conclusions from the self-evaluation. Nonetheless, the soft hand reached very good acceptability at SUS for 3 subjects (Fig. 3c), with a score close or higher than 68 (the average score in literature to consider a device accepted). On the contrary, commercial rigid devices never passed the acceptance criteria. In the TAPES-R*, rigid and soft devices reached comparable scores on the design satisfaction, even though the SHP is a research system under development. As suggested in^38^, TAPES-R should include two subscales inside of the satisfaction subsection, one for aesthetics and one for functional aspects. A unique value of satisfaction can mislead information about these two aspects, and a further investigation on the aesthetics should be included in future studies. In DASH, the soft hand obtained lower scores (better outcome) when participants perceived functional differences between prostheses. The authors believe that the participants’ promising attitude towards soft proprieties is related to their appreciation of the SHP intuitive use, soft interaction and adaptive capacity.

Despite the soft hand presents a closer resemblance to the healthy hand model in the self-location test (Fig. 4a), both soft and rigid prosthetic devices obtained similar finger lengths in absolute values (Fig. 4b). The smaller dimensions of the SHP compared to the i-limb could have influenced this finding. Regarding the radial-ulnar effect for prostheses, the thumb showed the strongest underestimation in Fig. 4d, especially for the rigid hand. This misperception could be influenced by the lack of opposition (or palm) in the design of both devices, making users perceive a shorter and less functional thumb than in human hands. Figure 4e shows a larger underestimation of the thumb-index knuckles compared to the literature (healthy subjects). This aspect reinforces the previous hypothesis of associating this misperception with a lack of anthropomorphism in the design or functionalities of the robotic thumbs. We observed an opposite effect of training for the two design solutions in Fig. 4f. This effect may occur due to the lack of movement in the transversal direction in rigid commercial prosthetic hands, which usually are designed to only favour fingers flexion/extension, neglecting the human capability of fingers adduction/abduction. However, the synergistic design of the SHP allows for a minimal transversal displacement of its fingers. This difference in the design could affect the perception of the hand width model and proves the actual visualization of the corresponding prosthesis during the experiment. Finally, in agreement with the literature, the aspect ratio of the human hands (i.e. *actual*) and the prosthetic devices (marked with dashed and dotted lines in Fig. 4g) showed larger fingers than the hand width, while all body models got a larger width than the perceived finger lengths.

During the sense of agency test, the poor adaptability and the need for compensatory movements of the rigid hand adversely affected the execution time in motor tasks (Fig. 4j). The considerable post-training improvement observed with the soft hand in Fig. 4l could indicate higher expertise acquired through the training session.

**Figure 6:**
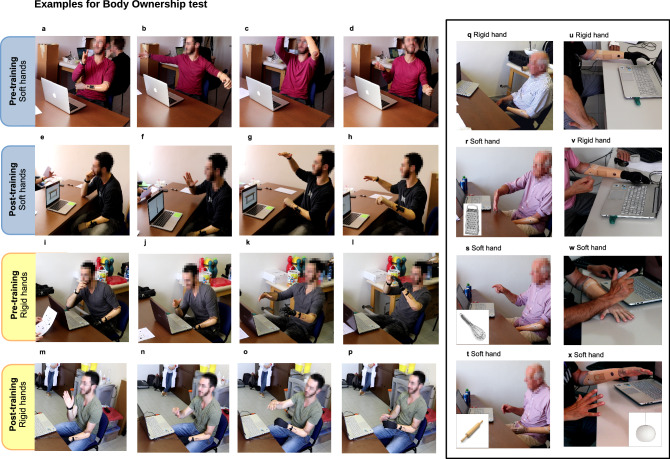
Body ownership test. (**a**–**p**) Show examples of the test executed by s_5_ in different conditions. (**q**–**t**) Present frames of the experiment with s_4_, while (**u**–**x**) with s_3_.

Figure 6a–p present some examples of the body ownership test with the most dexterous participant (Subject 5). Figure 6a shows a natural posture with the soft hand, where the prosthesis becomes part of user’s body image. The user gestured with his two hands to express mainly sizes (Fig. 6b) or complex object shapes (Fig. 6c). Only when using the soft hand, we observed the inclusion of the system for non-representative gestures (i.e. rhythmic gestures used to emphasize words and interactive gestures directed at the addressee) even in the pre-training condition (Fig. 6d), suggesting a higher preliminary sense of ownership. Contrarily, Fig. 6e, i and m show how for the soft hand post- and the rigid hand in both pre- and post-training conditions, the user rests the prosthesis on his leg, discouraging its inclusion. In these conditions, the participant mostly uses the intact hand to support the description of the objects (Fig. 6f, j and n) and uses both hands only for objects with higher dimensions (Fig. 6g, h, k and o). Figure 6l and p show the user gesturing with the rigid hand to describe the object shape or for non-verbal communication, respectively. Although the distinction among gestures was out of the scope of this study, we observed most of the representations defined in^39^ in this dexterous user, except for drawing. Contrary to our expectations, the results in Fig. 4n for Subject 5 suggest a lower gesture rate and a smaller variability with both devices in post-training conditions, which could be related to tiredness. Gestures are often the representation of a personal motor experience, and it was proved that speakers gestured more when describing actions manually executed previously^40,41^. It is possible that the condition of non-expert myo-electrical users has a strong effect on the participants’ gesture rates. Despite the body ownership test, designed by^39^, offers a systematic methodology, the authors believe that including objects used during the training could be beneficial to study this effect in prosthetics.

We present some interesting though anecdotal observations of the two other patients (Subject 4 and Subject 3). While Subject 4 never used the rigid hand to support the description of objects, he occasionally showed some movements with the soft hand. This could be related to several aspects, such as the difference in weight or encumbrance, but it may be a preliminary expression of the embodiment of the prosthesis. As shown in Fig. 6q, the subject did not use often the intact hand to communicate, and he did only after specific questions from the experimenter about the size of an object (Fig. 6r: Grater—manipulable object) or for an object that is difficult to verbally describe (Fig. 6s: Whisk—manipulable object). During the test with the soft hand, the user spontaneously used both hands to describe an action commonly executed (Fig. 6t: Rolling pin—manipulable object) for 5.33 s. These observations are supported by the experience of Subject 3. The participant did not use the rigid hand to support the description of objects either, and kept the device in a resting position (on the table) during the whole test (Fig. 6u). The subject only moved the intact hand during the experiment, but sometimes we observed involuntary changes of the i-limb grasping pattern (e.g. in Fig. 6v). Difficulties in controlling the system could discourage its spontaneous use. During the first day with the soft hand, the user included the prosthesis not only in his posture (Fig. 6w) but also in his body language to describe the shape of a light bulb (Fig. 6x—non-manipulable) for 3.77 s.

Overall, the results from the embodiment assessments showed a body model representation closer to reality and a larger number of spontaneous gestures when using the soft hand. Moreover, the results suggested a higher multi-tasking capability with the soft hand than with its rigid counterparts. These preliminary observations suggest a higher embodiment of soft properties and encourage future investigations in this matter.

## Material and methods

We designed an experimental protocol to compare the performance and user-perception of two myoelectric prostheses with different design features, one with soft and one with rigid properties. A group of non-expert myo-electric users tested the two devices in pre- and post-training conditions. Their naïve condition allows to observe not only their evolution throughout the training but also which features favour a more intuitive usage or a faster integration. All the participants tested both prosthetic systems, and we compared the results in a paired way.

### Ethics statement

This study was authorized by the local Ethics committee of Area Vasta Nord-Ovest (CEAVNO), Tuscany (Italy), protocol n. 7803. The clinical trial was conducted in the Operational Unit of Recovery and Functional Rehabilitation, USL Tuscany Nord-Ovest (Italy). Each patient recruited gave their written informed consent under the Declaration of Helsinki. All participants’ informed consent was obtained to publish the information/image(s) in an online open-access publication.

### Participants

**Figure 7:**
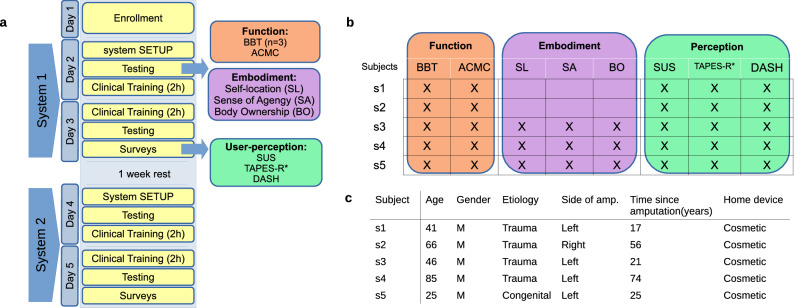
Experimental protocol. (**a**) Shows a schematic description of the experimental protocol. After the enrolment, each subject conducted 2 experimental sessions divided by a week break. Each session was devoted to the evaluation of one of the two systems (rigid and soft), usually comparing data from pre- and post-training. (**b**) Presents a table that marks the test and survey performed by each participant (s_i_) included in the study. (**c**) Gives the subjects demographics.

Five transradial amputee subjects were enrolled at the Recovery and Functional Rehabilitation Unit. Inclusion criteria were: (1) age between 18 and 85 years; (2) upper limb impairment related to limb loss; (3) ability to give informed consent. Subjects’ demographics at the time of evaluation are reported in Fig. 7c. Four subjects have a left-hand amputation and only one person presents a right-hand amputation. All participants are non-expert myo-electric users and regularly use a cosmetic prosthesis.

### Experimental setup

The experimental setup comprised a rigid and a soft artificial hand, controlled with 2 surface EMG sensors. As rigid hands, the i-Limb Ultra hand^5^ (Össur) and the Bebionic hand^4^ (Ottobock) were used by participants with a left side and right side amputation, respectively. The i-Limb Ultra has five individually powered digits and offers an electrically rotating thumb with a manual override. Likewise, the Bebionic hand has individual motors in each finger that coordinate for the execution of several grips. The soft hand tested is a research system named SoftHand Pro (SHP).

#### Soft system overview

The SHP is a prosthetic device with 19 DoFs controlled by one motor (see the system architecture in Fig. 1a). Designed through the concept of soft-synergies, the SHP integrates flexible roll-articular joints connected with elastic bands. Softness results especially useful and innovative for social interaction with others (e.g. Fig. 1c–d) and with the user’s own body (Fig. 1e–f). Figure 1g–h presents examples of coordinated bimanual tasks that are enhanced by its adaptive capability. Its softness increases the system robustness, which permits extreme finger configurations as in Fig. 1i–j with no risk of damage. With only one motor, its poly-articulated features and adaptive design allow the execution of multiple grasps by exploring the environment (e.g. Fig. 1k–n).

During the experiments, all prosthetic hands were connected to a passive rotational wrist (Hand Chassis with Quick Disconnect Wrist, Ottobock) for prono/supination hand orientation. A customized socket was fabricated for each subject with the help of a trained prosthetist. Two surface EMG sensors (13E200 MyoBock electrodes) were integrated into the socket to detect the electrical activity in the muscle and control the terminal device. Commercial poly-articular hands use different techniques (i.e. muscle co-contraction, long open activation, etc.) to switch between grip patterns. The control of the range of aperture/closure is usually with Proportional Velocity Control (PVC) (^42^ for a review), as the control of the SoftHand Pro. The therapist considered PVC a suitable control solution for all the participants. The subjects selected 4 of the automated grips for both commercial devices and defined the switching input modality according to their preferences with the help of a technician. Some of the most common grips chosen were fine pinch and three-digit grasp.

### Experimental protocol

After recruitment, the protocol includes 2 experimental sessions interspersed by a week break. Each session focuses on one system and comprises 2 consecutive working days of alternating training and testing. To avoid user’s risk of fatigue, we divided the therapeutic training into the two days. The presenting order of the systems (rigid/soft) was randomized among subjects. Figure 7a presents a schematic of the experimental protocol and the selected outcome measures. The experimental protocol was designed with the guidance of occupational therapists, involved also during the experimental sessions. Because of job requirements and time constraints, two subjects could not participate in the embodiment experiments (see Fig. 7b).

The protocol meets the following steps:

1. *Day 1* screening of the systems involved, followed by the enrolment of the patient. A therapist supervised this process to check participants’ medical history and collect informed consent. No detailed information was given about the capabilities of the systems under study not to influence their perception, neither create unrealistic expectations, as suggested in^43^.
2. The users answered a survey about general preferences and priorities in upper-limb prostheses (see Fig. 3a–b).
3. *Day 2* system training (e.g. calibration) and a short familiarization. The thresholds and gains inside of the control loop were customized and adjusted for the participant.
4. Right after, a pre-training testing phase was conducted. The user performed 2 standard assessments, the Box and Blocks test (BBT) and the Assessment for Capacity control of Myoelectric Control (ACMC), and 3 customized tests regarding embodiment.
5. Then, the user trained for 2 h with the therapist. Prosthetic training is a dynamic process that includes orientation, control, use, and ultimately, activities of daily living^44^.
6. *Day 3* Starts with 2 h training, following what participants learned the previous day. The training activities slightly changed depending on the individual response and level of dexterity of the participants.
7. Then, post-training testing was conducted, following the same activities of step (4).
8. Once the evaluation of a system is completed, the user responded to three standard outcome surveys (SUS, TAPES-R* and DASH) accounting for user-perception.
9. *Day 4 and 5* The same process described in steps (3–8) was repeated with the other system after a week of rest.

More details can be found in the media attachment (S1) and in Supplementary Material and “Material and methods” section. The heaviest object grasped during the experimental protocol weights 1 kg (during the ACMC test).

### Functional assessment

The users performed two standard assessments to evaluate the hand functionality: the Box and Blocks test (BBT) and the Assessment for Capacity control of Myoelectric Control (ACMC).

The BBT measures unilateral gross manual dexterity^45^ and it is used with a wide range of populations (i.e. not specific for prosthetics). The user has to move the maximum number of blocks, one by one, from one compartment to another within 60 s (see an example in Fig. 4a). We repeated this action 3 times. To evaluate advanced functionalities and manipulation skills, we included the ACMC test. It is the first outcome assessment specifically designed for myo-electric prostheses. The ACMC is an observational-based assessment measuring a person’s ability to operate a myo-electric prosthetic hand when performing ordinary life activities^46^, as seen in Fig. 4b. The test comprises 22 items divided into 4 subsections (Gripping, Re-adjusting Grip, Holding, Releasing) and scored on a 4-point rating scale by a certified observer. Items differ in movement and level of difficulty. In this protocol, 4 tasks were selected and randomized among participants, sessions and devices. These 4 tasks are: setting of the table for breakfast, preparing a cake, packing of a suitcase and the organization of a post box. It is recommended to avoid task repetition to keep the subject motivated and to accomplish the tasks spontaneously in their usual way.

In^46^, the ACMC proved to be a valid, reliable and sensitive evaluation of qualitative aspects of myo-electric control and hand features. It observes changes over time and especially when the user is not an expert. For this reason, the choice of naïve users creates a unique opportunity to highlight important aspects related to the prosthetic devices and their effect on subjects’ learning.

### User-perception

Large changes in manual dexterity skills can have a different effect on client-rating measures. For this reason, it is fundamental to consider different aspects in the self-evaluation of an experiment^47^. We asked the user to complete the SUS, TAPES-R* and DASH surveys at the end of each system session.

The system usability scale (SUS)^48^ gives information about the acceptability and usability of a system. TAPES-R^38^ is a survey widely used in lower-limb amputees and related to the impact of the device usage on the subject’s quality of life. It was theoretically and empirically derived to enable an examination of the psychosocial processes involved in adjusting to amputation and the specific demands of wearing a prosthesis^49^. Since this study involves naïve users, we considered only the subsection regarding perceived satisfaction, and we added an asterisk to the survey name. Note that from the standard TAPES-R measure, the explored subsection only allows a maximum score of 12. The DASH^50^ is commonly used for patients with upper-limb disabilities and evaluates the hand functionalities perceived by the user. The user is asked to assign a difficulty score (from *no difficulty* 0 to *incapable* 4) for specific daily living actions performed with the evaluated system. This test includes 2 optional sections about work and sport/music abilities.

### Embodiment assessment

Prostheses are necessary aids to recover normal appearance and functional independence, but little is known about the requirements for a good embodied experience. While this aspect is fundamental for the users’ acceptance, no standard assessment faces this evaluation given its complexity and poor understanding. In^51^, Murray highlighted the importance of this aspect and the involvement of several interrelated areas. Embodiment is described as the capability of something to become incorporated into the phenomenal boundaries of the body, i.e. a phenomenological osmosis^52^. It is accepted that external systems could extend the realm of the senses and withdraw into the sensorium of the body. Therefore, it is fundamental to transform a prosthetic limb from an inert supplement or an extracorporeal structure into a corporeal one^53^. The identification of the process and steps necessary to achieve this experience could allow improvements of the rehabilitative process and the development of the properties and functional features required by subjects in their bionic aids. Even with a small group of users, the authors considered it essential to explore concepts and theories about embodiment, and study potential effects on naïve users. We focused on three components describing the sense of embodiment^36^: (a) the self-location, (b) the sense of agency and (c) the body ownership. We selected 3 related tests from the literature that could be applied to prosthetics and conducted the study with 3 (male) subjects using a rigid (i-limb) and a soft (SHP) hand.

Knowing the body's location in external space is a fundamental perceptual task, but no sensory signal is directly informative about the size and shape of our extremities. Perceiving our body location is essential for interacting with the environment, and a high distorted representation of our hand could hinder advanced manipulation skills. We can not underestimate this issue in prostheses, as users integrate an artificial system as part of their body and it should be incorporated in their body model for appropriate usage. For this reason, we evaluate a related test called self-location. In^37^, the authors tested the existence of a stored body model based on metric properties (i.e. body part size and shape) through the recognition of 10 landmarks on the hand in able-bodied subjects. The distance between the judged locations of two adjacent landmarks (e.g. the tip and knuckle of a single finger) depends only on the represented length of the body segment connecting them. This psychomorphometric approach focuses on the perception of hand shape and offers a quantitative method to study an elementary form of self-awareness in the brain. In this work, we apply the same method to evaluate the perception of each prosthetic device before and after training. As shown in Fig. 4h, we asked subjects to accommodate the hand inside a box positioned on a table. Then, they marked with a pen the presumed location of the 12 landmarks of the concealed hand on a graph paper located over the box. The position of various landmarks serves to build a spatial map of the patient’s hand model and we contrast the results with those of their intact hand. Figure 4h shows the experimental setup and a representation of landmarks to be drawn for each hand of study. For our study, the percentage of overestimation was calculated as (100 × judged length-actual length)/actual length. The shape index was defined as 100 × (width/length) and calculated for user’s healthy hand, both prosthetic systems and the anthropomorphic values of the healthy hand (i.e. actual). The length of the middle finger and the distance between index-little finger knuckles were used.

The sense of agency is described as the consciousness of being the initiator of a body action^54^. To study this aspect, we evaluate the simultaneous performance of a simple manipulation task combined with a cognitive task. The cognitive task consists in the counting of a random number of beeps that occur in a maximum of 60 s. Whereas the manipulation task (Fig. 4i) consisted in either one of two different actions: moving 5 cans from one position to another and turning 5 cards upside down. Inspired by the Jebsen–Taylor Test, five cans (height, 9.5 cm; diameter 7.5 cm) with two different weights (weighting < 20 g and 0.45 kg, respectively) were randomly placed in front of the board. Subjects should lose the focal awareness of the prosthetic aid and use it as a practical extension of their body. We hypothesize that hands with a higher embodiment allow the user to execute accurately the counting action while being faster in accomplishing the manipulation action.

To assess body ownership, we considered the involvement of the prosthetic hand in spontaneous activities. Several studies have focused on different aspects of self-expression and gestures, which are strongly related to the sense of embodiment^55^. Gestures occur often with content that evokes imaginary^56^ or when speakers reflect events that emphasize spatial and motoric information^57^. In^39^, the authors designed a systematic method that showed a connection between object characteristics and representation techniques in spontaneous gesture production. Results in^39^ suggested that embodiment could be related to the functionality/dexterity perceived by the subjects and the capability to imagine themselves producing such actions. Inspired by^39^, our experiment consists in presenting images (displayed on a laptop screen) of several objects in a randomized order. The prosthesis user is asked to describe the objects to a listener, as if this one should go later to a department store and buy them. The listener should be able to visualize the objects in a paper brochure, in which all objects appeared forming a grid. It is possible that intensive training or specific hand properties influence a higher capability to perform body language.

### Statistical analysis

The acquired data were analyzed in MATLAB (MathWorks). All data were reported as mean values ± SD (unless elsewise indicated). For functional assessment and embodiment evaluation, n-Way analysis of variance (ANOVA) test was applied to include different factors that may influence their results. Post hoc correction was executed for multiple groups of data, specifically a Tukey test. We applied paired-sample t-test for surveys comparing rigid and soft hand scores. Significance levels were 0.05. In the captions of the figures, we reported the used statistical tests for each analysis and its result.

## Supplementary Information

Supplementary Video 1.

Supplementary Video 2.

Supplementary Information.

## Data Availability

All relevant data are within the paper.
