# Supplementary material for: Comparison between rigid and soft poly-articulated prosthetic hands in non-expert myo-electric users shows advantages of soft robotics
Source: Sci Rep. 2021 Dec 14;11:23952. doi: 10.1038/s41598-021-02562-y (PMC8671573; doi:10.1038/s41598-021-02562-y)
Supplement: Supplementary file 3 — Supplementary Information. [file 41598_2021_2562_MOESM3_ESM.docx]

**Supplementary Material:**

**Supplementary Materials**

Materials and Methods

Discussion

Supplementary References

Figure S1

Movie S1. Experimental Protocol.

Movie S2. Training Protocol.

Material and Methods

**Training protocol**

New physical and informational properties of a prosthetic hand must be internalized to use them adequately (*S1*). We hypothesize that awareness of the functional properties of a hand and appropriate training can favour hand usability and spontaneity. Occupational therapists provide training in natural contexts so that competence in everyday life use can be ensured. The key aspects included in the training sessions are the following. More details can be found in the media attachment (S2).

- Muscle activation; patients were instructed to activate the muscles that are needed for myoelectric control. The therapist showed to the patient the effects of muscle contraction on the sound limb and then repeated this process on the amputated arm. The goal is to increase muscle strength and isolate muscle contractions.
- Mirror therapy; to favour embodiment and coordination, the user practice synchronous and asynchronous movements of both hands.
- Control coordination; the training focused on the timing between hand opening and hand closing, paying attention to synchronized and coordinated movement. Control training teaches proficiency in the mechanical operation of the prosthesis.
- Manipulation skills; training progress from gross-motor activities to fine-motor and manipulative activities. Initially, training included simple grasp and release activities using hard objects with geometrical shapes. The object release progress from simple actions to aiming for a specific target. Then, patients are instructed on stabilizing objects with the prosthetic hand and trained with objects of different sizes and stiffness.
- Multiple-joint coordination; treatment focused on the control of proximal joints, which is needed for proficient use of the entire arm. Activities include simple grasping and releasing at different heights and arm positions. Therapists also promote the use of the prosthesis while moving the arms in the space and body perception to address users’ daily life problems.
- ADL; treatment protocol included prosthetic usage based on activities of daily living (ADL), which requires applying the learned skills into functional activities.

Discussion

**Additional observations**

The authors reviewed all the video footage of the training sessions and include additional observations in this section.

The subjects interacted with the prosthesis during breaks, and the complexity level of their interaction increases with the patients’ learning. While for rigid hands subjects interact mostly with the passive joints of the prostheses, as occurred with the thumb abduction/adduction of the i-limb (Fig. S1a), soft technologies seem to promote a faster and natural inclusion of the overall artificial hand (Fig. S1b). We observed that users essentially include the prosthesis into their speech when speaking about the cause of the limb loss, but without active use of the hand (see Fig. S1c-d). The limited use (only 4h training) may be deficient for the prosthesis inclusion into users’ body language.

Users performed faster and with less compensatory movements with the soft hand when power grasps were involved. However, when a precision grip was demanded, the improvement compared with the rigid hands was less evident. While rigid hands provide accurate precision grasps, the pinch in the SoftHand Pro occurs in an intermediate position of the total closure of the hand. This issue is emphasized when larger applied forces are required (e.g. grasping heavy objects). However, as human fingers adapt with the contact of the target to perform an appropriate pinch, we believe that a compliant hand, where the rigidity of the fingertips or the hand can be controlled, could be an interesting solution. A preliminary investigation of this is proposed in (*S2*). These observations also suggest the study of alternative design solutions to extend the grasping capabilities of the SHP, as proposed in (*34*).

Because of the reduced number of DoFs and rigid connections in the i-limb or Bebionic prostheses, these hands presented grasps with few contact points and objects often fell during holding. Regarding the release phase, the subjects often encountered more difficulties in a stable release after a precision grasp with the SHP. However, its adaptability allows adjusting slightly the orientation of objects without the reopening of the hand (i.e. in-hand manipulation). Sometimes the participants may benefit from using the passive rotational wrist, no matter the prosthesis used, to avoid excessive compensatory movements. However, they usually tried to complete the task without its use, and only when they found major difficulties, they used their intact hand to prono-supinate the artificial hand. This highlights the complexity in body trajectory prediction when there is insufficient practice.

Subjects sometimes experience difficulties in controlling their prostheses, which lead to poor reliability on the device and disfavours the voluntary use of the robotic aid in ADL. The residual muscle activity, the level of training, the fatigue and the fitting of the socket are reasons that affect the myo-electric control. We noticed that subjects were forced to stop the action and support the socket in many situations to assure good contact between the skin and the EMG sensors (see Fig. S1e-f). Despite the therapist encourages the use of different grasp patterns in rigid poly-articulated hands, we did not observe voluntary switching in goal-oriented actions, with preference for power grasp. Most of the subjects experienced undesired switching (see an example in Fig. S1g-h) when moving the arm in space or due to involuntary muscle activation. Non-expert myo-electric users probably require intensive training for the effective incorporation of switching techniques. Fig. 8i-l show examples where the subjects passed some weights through a pencil or a string. The need for supporting the prosthesis with the intact hand (Fig. S1j) compromises the coordination of both arms. See the photo-sequence in Fig. S1k-l for a successful execution of the task with the soft hand.

Supplementary References

S1. Churcher, J. Implications and applications of piaget’s sensorimotor concepts. In *Adaptive Control of Ill-Defined Systems*, 289–304 (Springer, 1984).

S2. Capsi-Morales, P., Piazza, C., Catalano, M. G., Bicchi, A. & Grioli, G. Exploring stiffness modulation in prosthetic hands and its perceived function in manipulation and social interaction. *Front. Neurorobotics* **14**, 33 (2020).


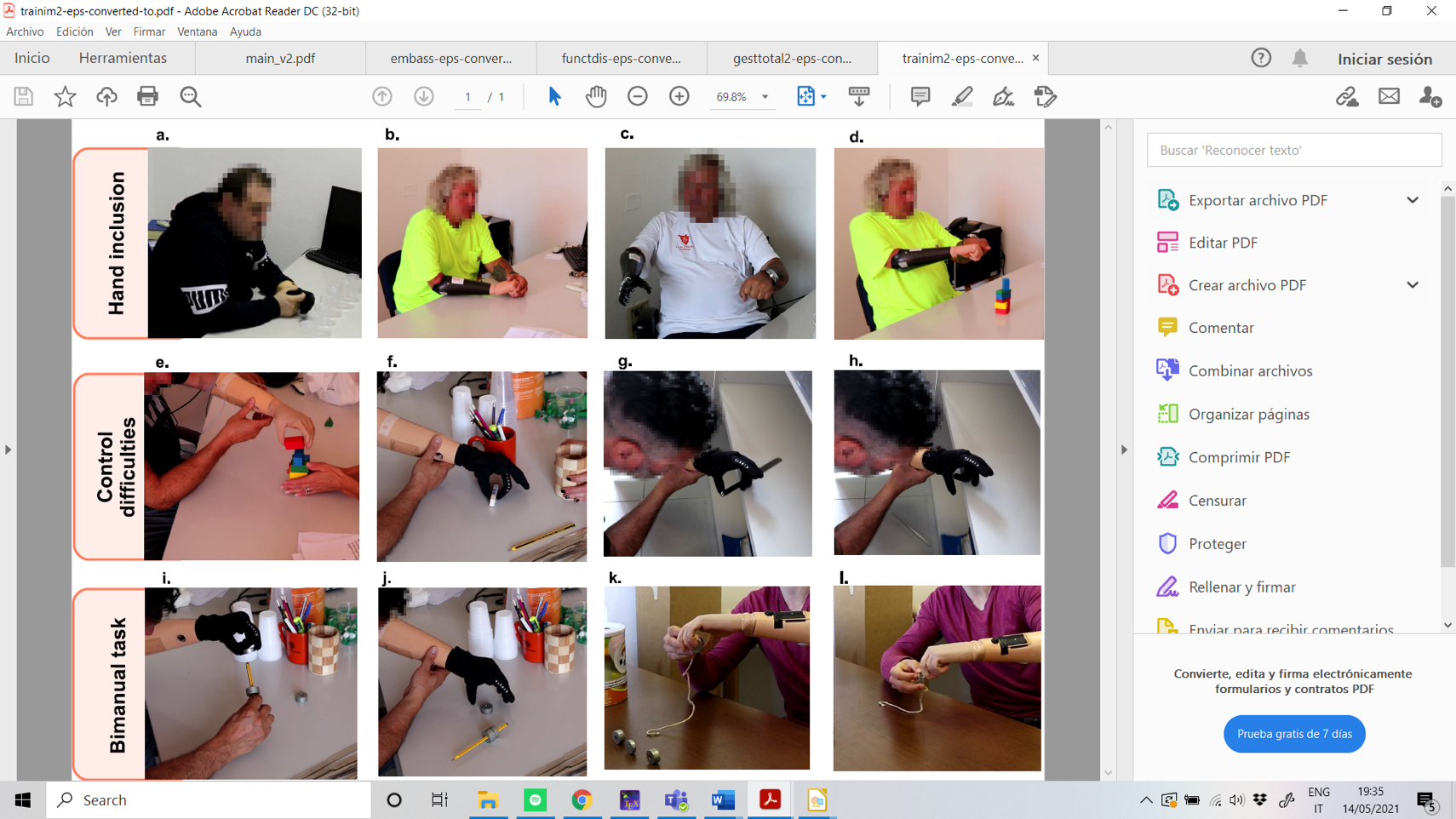


**Fig. S1.** **Additional observations. (A-D)** shows natural interaction with the prostheses; **(E-H)** some myoelectric control difficulties in poly-articulated hands and **(I-L)** bimanual manipulation capabilities.
